# Microvascular reconstruction for maxillofacial defects: a retrospective analysis of outcomes and complications in 121 consecutive cases

**DOI:** 10.1186/s40902-020-00273-4

**Published:** 2020-08-26

**Authors:** SeongRyoung Kim, Dong-Hun Lee, Kang-Min Ahn

**Affiliations:** Department of Oral and Maxillofacial Surgery, College of Medicine, University of Ulsan, Asan Medical Center, 88 Olympic-ro, 43-gil, Songpa-gu, Seoul, 05505 Korea

## Abstract

**Background:**

Microvascular reconstruction is the treatment of choice after oral cancer ablation surgery. There are few published studies of free flap survival among Korean populations. This study aimed to determine the survival rate after 121 consecutive cases of maxillofacial microvascular reconstruction and to analyze the complications associated with microsurgery.

**Methods:**

This study included consecutive patients who underwent microsurgical reconstruction with free flaps, from January 2006 through September 2019, performed by a single surgeon at the oral and maxillofacial surgery department of a tertiary medical center. A total of 121 cases were reviewed retrospectively. The flap survival rate, flap type, radiotherapy history, complications, and treatment results were analyzed.

**Results:**

Four different flap types were used for microvascular reconstruction: radial forearm (*n* = 65), fibula (*n* = 34), latissimus dorsi (*n* = 21), and serratus anterior muscle with rib bone free flap (*n* = 1). Total necrosis of the flap was found in four cases (two latissimus dorsi flaps and two fibular flaps). The free flap survival rate was 97.5%. Nineteen patients received radiotherapy before surgery, and none of them experienced flap failure. The mean operation time was 334 ± 83.1 min, and the mean ischemic time was 48.9 ± 12.7 min.

**Conclusions:**

The success rate was reliable and comparable with previous studies. The success rate was not affected by radiation therapy. Free flaps can be safely used even after radiation treatment.

## Background

Microsurgical reconstruction of the oral and maxillofacial area is a challenging procedure that involves pedicled flaps [1–8]. Reconstructive surgeons should be thoroughly familiar with the anatomy of the donor site and be able to dissect neck vessels and restore damaged structures with available flaps to produce favorable functional and esthetic outcomes.

With improvements in microsurgical techniques and instruments, free tissue transfer has become the most reliable method for treating head and neck defects. The free flap technique represents a revolution in reconstructive surgery, as it enables the harvesting of a large amount of revascularized tissue, which can be tailored to the defect and allow for more complex reconstructive procedures, while simultaneously permitting more extensive head and neck resections [9–11]. Different free tissue flaps have been reported as conducive to the reconstruction of head and neck tumor defects. These include latissimus dorsi (LD) flaps, radial forearm (RF) flaps, scapula flaps, anterolateral thigh flaps, jejunum flaps, and rectus abdominis muscle flaps [12–15].

Free flaps have been widely used in Korea after oral cancer and reconstructive surgery; however, there are few reports about flap survival and clinical results of free flap reconstruction [6]. This retrospective study investigated the survival rate, complications, and outcomes of 121 consecutive microsurgical reconstruction cases in a single institution by one experienced surgeon.

## Methods

This study included patients who underwent microsurgical reconstruction with free flaps, from January 2006 through September 2019, performed by a single experienced surgeon at the oral and maxillofacial surgery department of a tertiary medical center. A total of 121 cases were retrospectively reviewed. The flap survival rate, flap type, complications, and treatment results were captured and analyzed. The study protocol was reviewed and approved by the institutional review board of the Asan Medical Center (IRB approval No. 2019-0197).

Microvascular anastomoses were mainly performed using the superior thyroid artery, facial artery, and external carotid artery. Simple interrupted suturing was performed using 9-0 or 10-0 nylon (Ethicon, J&J Medical Devices, England) sutures. Intraoperatively, a heparin–saline was applied to donor-site and recipient-site blood vessels to prevent vascular coagulation. Doppler monitor was used to confirm the state of the free flap until 7 days after the operation.

The characteristics of patients including primary disease, types of flaps, operation time, ischemic time, and post- and pre-operation radiotherapy were reviewed. The survival of free flap was determined by clinical evaluation in cases of soft tissue flaps and bone scintigraphy in bone flaps. The complications associated with free flap reconstruction were examined retrospectively.

All statistical analyses were performed using SPSS Statistics for Windows, version 21 (IBM Corp., Armonk, NY, USA). Results were presented as either means ± standard deviations or frequencies with percentages. A *p* value < 0.05 was considered statistically significant.

## Results

**Fig. 1:**
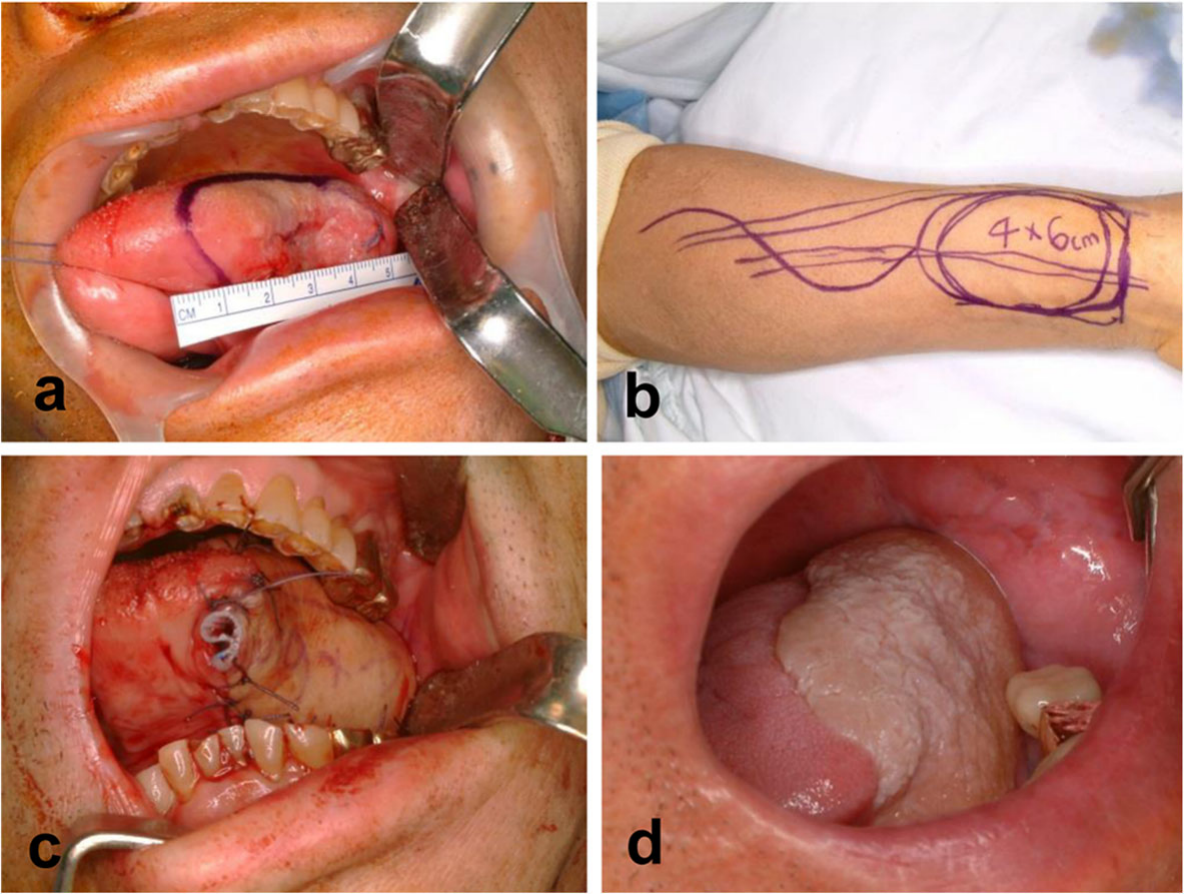
Surgical approach and clinical results after radial forearm free flap (**a** left tongue cancer, **b** radial forearm free flap design, **c** radial forearm free flap reconstruction of the lateral tongue, **d** tongue reconstruction 3 years after operation)

**Fig. 2:**
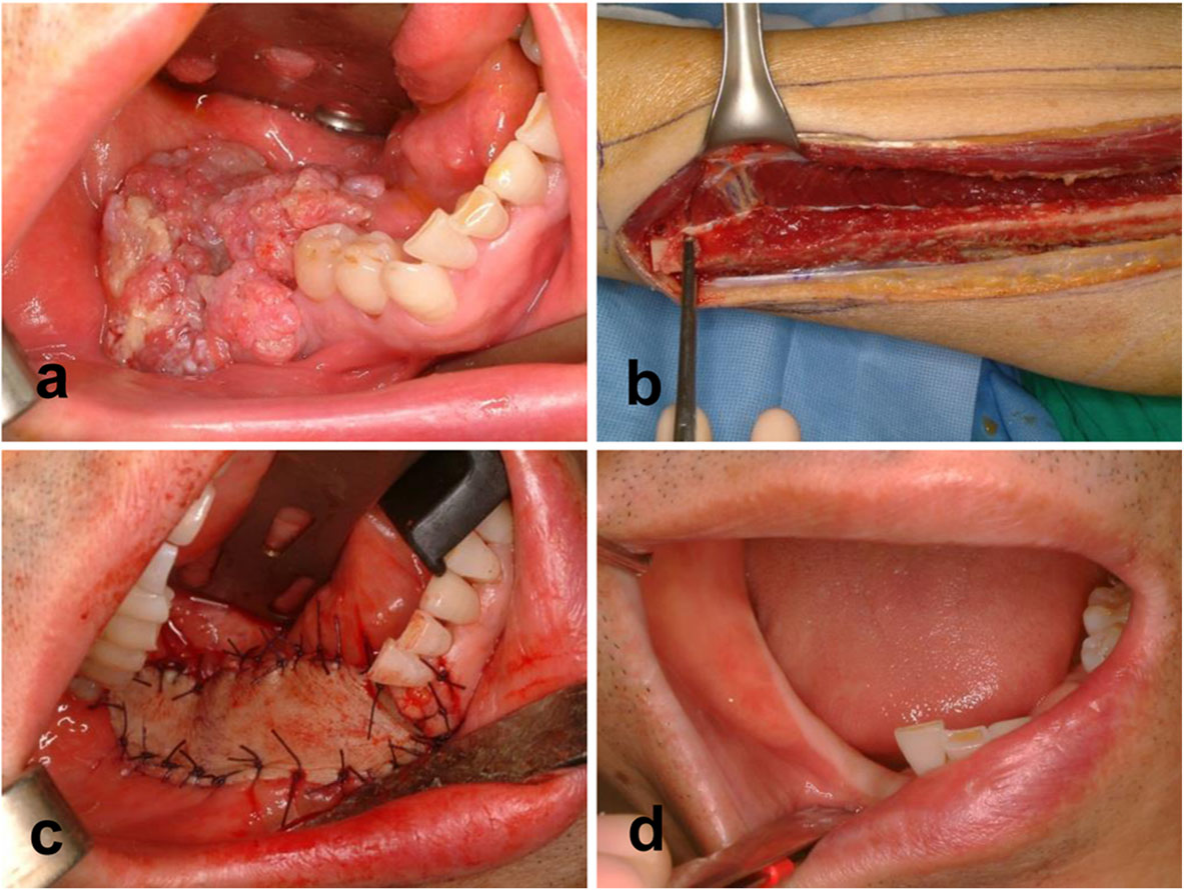
Surgical approach and clinical results after fibular free flap (**a** right mandible gingival cancer, **b** fibular free flap elevation, **c** intraoral position of skin paddle, **d** intraoral photograph 3 years after operation)

**Fig. 3:**
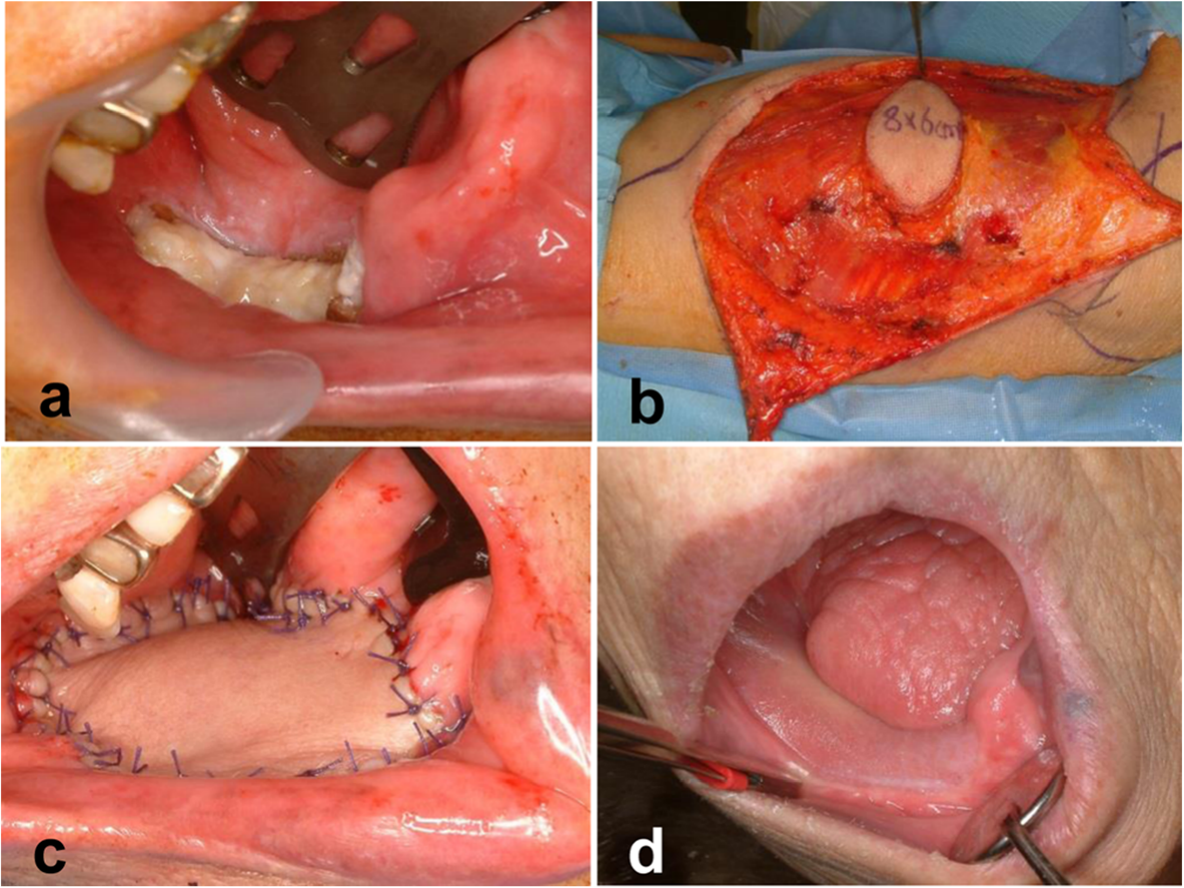
Surgical approach and clinical results after latissimus dorsi flap (**a** right gingival cancer with bone invasion, **b** latissimus dorsi flap elevation, **c** intraoral position of skin paddle, **d** intraoral photograph 3 years after operation)

**Fig. 4:**
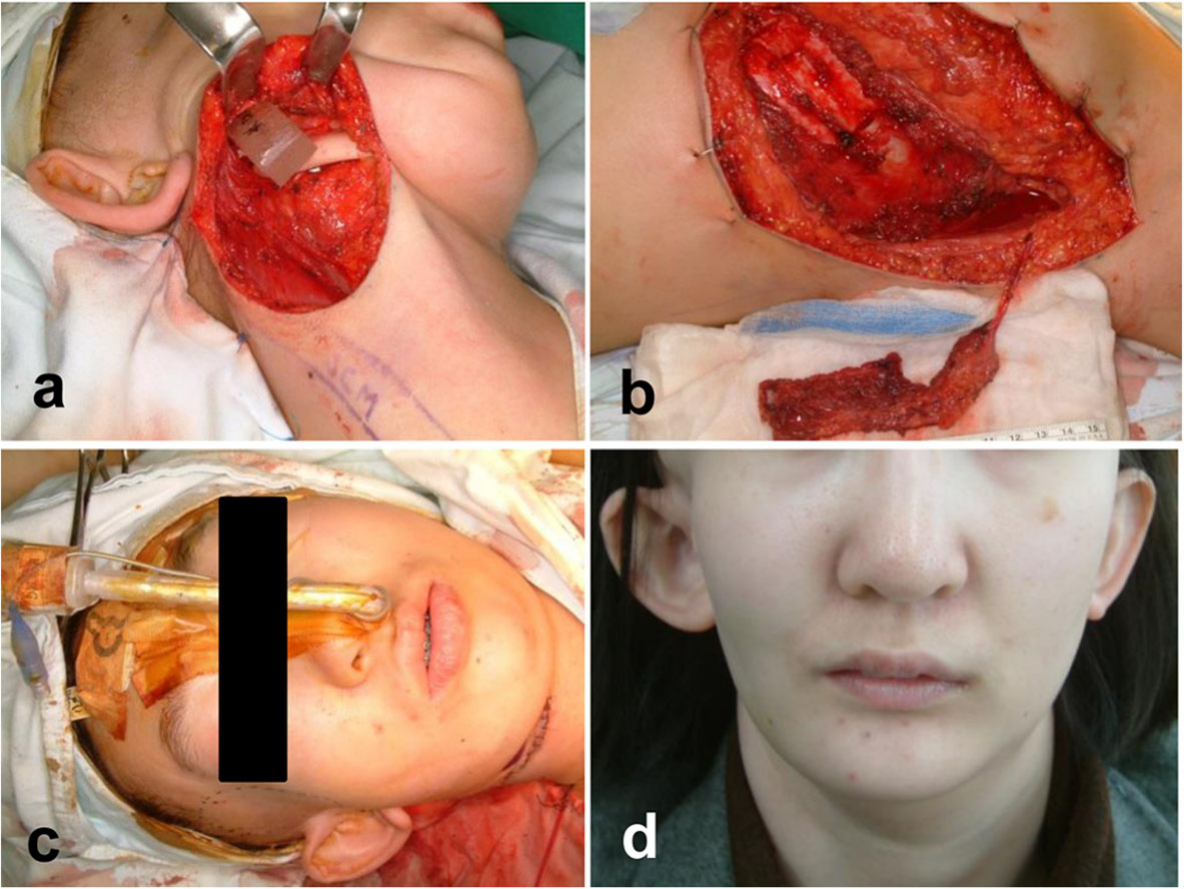
Surgical approach and clinical results after serratus anterior with rib bone free flap (**a** exposed right mandible, **b** elevated flap with rib bone, **c** intraoperative photograph for mandible reconstruction, **d** facial photograph 5 years after operation)

Table 1 reports the characteristics of the included patients and Figs. 1, 2, 3, and 4 shows the representative case of reconstructed flaps. There were no significant differences in sex ratio or mean age between the groups of each flap (*p* = 0.64 and *p* = 0.25, respectively). Of the 121 patients, 12 had postoperative complications regarding free flap reconstruction (Table 2). The flap types and the postoperative outcomes are shown in Table 3. Total necrosis of the flap was found in 4 cases (two latissimus dorsi (LD) flap and two fibular flaps) and the failed flaps were debrided. Two LD flaps were reconstructed with the opposite side LD flap. Two failed fibular flaps were debrided and defect sites were healed with secondary intention. The overall survival rate of the free flaps was 97.5%.

**Table 1:** Patient characteristics

	RFFF	FFF	LD	SA	Total	*p* value
Gender, *n* (%)						0.64^a^
Male	43 (66.2)	17 (50)	15 (71.4)	0 (0)	75	
Female	22 (33.8)	17 (50)	6 (28.6)	1 (100)	46	
Age, mean (SD) (years)	60.3 (13.4)	58.4 (12.2)	59.2 (12.1)	13.00 (0)	59.2 (13.4)	0.25^b^

*SD* standard deviation, *RFFF* radial forearm free flap, *FFF* fibula free flap, LD latissimus dorsi, *SA* serratus anterior

^a^Linear-by-linear association

^b^Kruskal–Wallis test

**Table 2:** Prognostic factors and outcomes

Factor	RFFF	FFF	LD	SA	Number of flaps	Flap loss
**Complications**						
Arterial thrombosis	0	0	2	0	2	2
Venous thrombosis	0	2	0	0	2	2
Fistula	1	2	0	0	3	0
Postoperative bleeding	1	0	2	0	3	0
Seroma	1	1	0	0	2	0
Total	3	5	4	0	12	4
**Previous radiation therapy**	7	8	4	0	19	0

**Table 3:** Type of flaps and postoperative outcomes

Type of flap	Cases (*n*)	Failures (*n*)	Success rate (%)
RFFF	65	0	100
FFF	34	2	94.12
LD	21	2	90.48
SA	1	0	100
Total	121	4	97.5

*RFFF* radial forearm free flap, *FFF* fibula free flap, *LD* latissimus dorsi, *SA* serratus anterior

The main indication for microsurgical reconstruction was the defect after cancer ablation surgery. The indications for flap reconstruction were listed in Table 4. The mean operation time was varied from 270.6 ± 50.3 to 400.1 min in each flap (Table 5). The mean ischemic time which refers to the time taken during microvascular anastomosis was less than 62.5 ± 5 minutes.

**Table 4:** Indications for microsurgical reconstruction

	RFFF (*n*)	FFF (*n*)	LD (*n*)	SA (*n*)	Total	%
Squamous cell carcinoma	57	18	14		89	73.6
Osteoradionecrosis	2	4	1		7	5.8
BRONJ		5			5	4.1
Adenoid cystic carcinoma	2	1			3	2.5
Osteosarcoma		2	1		3	2.5
Ameloblastic carcinoma	1		1		2	1.7
HCC metastasis		1	1		2	1.7
Mucoepidermoid carcinoma	1	1			2	1.7
Spindle cell sarcoma	1		1		2	1.7
Ameloblastoma		1			1	0.8
Flap necrosis			1		1	0.8
Parotid tumor		1			1	0.8
Postoperation bleeding			1		1	0.8
Rhabdomyosarcoma				1	1	0.8
Verrucous carcinoma	1				1	0.8
Total	65	34	21	1	121	100

*RFFF* radial forearm free flap, *FFF* fibula free flap, *LD* latissimus dorsi, *SA* serratus anterior, *BRONJ* bisphosphonate-related osteonecrosis of the jaw, *HCC* hepatocellular carcinoma

**Table 5:** Ischemic time and surgical procedure time for free flap reconstruction

	RFFF		FFF		LD		SA		Total	
	Mean	± SD	Mean	± SD	Mean	± SD	Mean	± SD	Mean	± SD
Ischemic time (min)	36.3	7.5	47.5	9.6	62.5	5	52.5	-	48.9	12.7
Operation time (min)	270.6	50.3	345.3	70.4	381.8	91.3	400.1	-	334.0	83.1

*SD* standard deviation, *RFFF* radial forearm free flap, *FFF* fibula free flap, *LD* latissimus dorsi, *SA* serratus anterior

## Discussion

The success rate of microvascular free flap reconstruction has risen from 70 to 90–99% over the past 30 years, making it the most reliable surgery in the field of reconstructive surgery [16–19]. In our retrospective study, the overall flap success rate was 97.5% among 121 consecutive procedures (Table 3). In our department, all of these procedures were performed by one oral and maxillofacial surgeon for 14 years. Therefore, our surgical outcomes have benefited from continuity, as we avoided the impact of differences in skill levels among multiple surgeons or centers.

Few studies investigating operating times for microvascular reconstruction have been published. It has been reported that as the operation time increases, the level of risk increases, particularly in terms of the incidence of rhabdomyolysis, fluid and electrolyte disturbances, deep vein thrombosis, and hypothermia [20, 21]. According to various reports [22–25], operation times vary from 376 to 817 min for maxillofacial microvascular reconstruction**.** Crawley et al. [26] reported a mean ischemic time of 115.4 ± 35.7 min among 849 cases and a mean operation time of 732 ± 144 min among 650 cases. They also found that prolonged ischemic times were associated with a higher flap failure rate. In our study, the mean operation time was 334 ± 83.1 min, and the mean ischemic time was 48.9 ± 12.7 min, meaning that our procedures were relatively short overall (Table 4).

Our team used 1-team approach for mass excision and microsurgical reconstruction. Theoretically, 2-team approach can reduce operative time and operator fatigue. However, the operation time may be increased due to team change during operation. Torabi et al. [27] compared data from 1- and 2-team performing head and neck reconstructive surgery in 2968 patients and reported longer operative time and more complication rates in 2-team approach. Reduction of operation time during microvascular reconstruction can minimize the surgeon’s strain and fatigue as well as reduce the risk of postoperative complications such as wound infections, hematomas, seromas, and dehiscence. The surgeon’s factor such as excellent microsurgical technique and experiences are important determinants for successful reconstruction [28].

Preoperative radiotherapy has been reported to be associated with a higher risk of free flap failure and complications [29]. In fact, radiotherapy causes microscopic and macroscopic vascular changes [30–32]. In our study, however, the 19 patients who had previously undergone radiation therapy had a 100% survival rate. Gordin and Ducic [33] reported that microvascular surgery could be successful among patients who have received multiple courses of preoperative radiation. It is clear that patients undergoing radiation therapy at the surgical site have an increased risk of postoperative complications. However, this disadvantage can be overcome by careful selection of blood vessels, with considerations such as adequate vessel diameter and vessels contralateral to the side of the body that was irradiated.

Anatomical limitations, such as vessel depletion, can increase operation times or make it difficult to find reliable recipient blood vessels. However, Nahabedian et al. [34] reported no correlation between the rates of flap necrosis and the choice of recipient vessels, the use of an interposition vein graft, the method of venous or arterial anastomosis, or the timing of reconstruction. In our retrospective study, four patients experienced total flap loss, but we did not observe any primary vessel depletion intraoperatively.

Some authors [35, 36] have reported that age alone should not be considered an independent risk factor or contraindication when considering free flap transfer. Ferrari et al. [35] reported a 98.2% free flap success rate among patients over 75 years of age and a 96.2% success rate among patients under 75 years of age. Tarsitano et al. [36] described the free flap success rate to be similar among patients over 75 years of age compared with the general population. In our study, we observed a mean age of 59.2 years (range, 18 to 84 years), with flap loss occurring in one of seven patients over 75 years.

In head and neck microvascular reconstruction, flap failure is usually caused by vascular thrombosis [37, 38]. Of the 121 cases in this study, flap failure occurred in 2 cases in LD flap and 2 cases in fibula free flap. In case of LD flap failure, vessel kinking occurred due to bulky flap size, resulting in arterial flow deficiency. In one of the fibula flap fail cases, flap necrosis happened after RT. It might be related with vessels damage due to high dose of RT. The other case failed due to venous thrombosis. Common causes of venous thrombosis are vasospasm caused by hypothermia, hypotension, and mechanical stress during anastomosis [39]. On the other hand, arterial thrombosis is caused by vessel kinking, bleeding, and extrinsic compression [40]. One patient experienced postoperative bleeding with RFFF. During bleeding control and debridement, the surgical defect became larger than the first surgery and neck vessels were exposed. In order to protect the carotid artery with a thick muscle layer, the existing flap was removed and LD flap was performed.

All patients were monitored immediately after reconstruction surgery. Experienced surgeon checked flap color and temperature every 8 h for the first 72 h. The risk of thrombosis is the highest at 80% by postoperative day 2 and decreases by 10% after 3 postoperative days [41]. Arterial crises manifest as pale flaps, capillary refill longer than 1 s, a lack of bleeding after a pinprick, and low flap temperature.

## Conclusions

Microsurgical reconstruction is the most versatile method for restoring large defects in the head and neck, and it has major implications in terms of patient quality of life. The success rate in our series was high at 97.5%. Microvascular free tissue transfer can also be successfully performed for patients who have undergone radiation therapy. Complications should be prevented by using a meticulous surgical technique and performing careful postoperative monitoring.

## Data Availability

All the data were retrieved from electronic medical chart of Asan Medical Center.
